# Overview of frequent pattern mining

**DOI:** 10.5808/gi.22074

**Published:** 2022-12-30

**Authors:** Jurg Ott, Taesung Park

**Affiliations:** 1 Laboratory of Statistical Genetics, Rockefeller University, New York, NY 10065, USA; 2 Department of Statistics, Seoul National University, Seoul 08826, Korea

**Keywords:** data mining, genotype pattern, machine learning, pattern recognition, statistical significance

## Abstract

Various methods of frequent pattern mining have been applied to genetic problems, specifically, to the combined association of two genotypes (a genotype pattern, or diplotype) at different DNA variants with disease. These methods have the ability to come up with a selection of genotype patterns that are more common in affected than unaffected individuals, and the assessment of statistical significance for these selected patterns poses some unique problems, which are briefly outlined here.

## Introduction

Frequent itemset (or pattern) mining (FPM) is now a well-established field with a rich literature and availability of software [1]. Here we loosely define a pattern as a sequence of specific items of interest with a specific frequency different from that expected by chance. Our primary interest, though not exclusively, is the pattern of DNA variants or genotypes associated with genetic diseases. Data mining is a broad field of research, and a major challenge in this field is pattern recognition or mining [2]. This introductory section briefly reviews some advances in human disease genetics, then sets out specifically to pattern recognition methods.

It has been known that many human traits follow Mendelian mode of inheritance and are passed from parent to offspring in a dominant or recessive manner [3]. However, most complex traits, such as diabetes, are influenced by multiple DNA mutations [4] and environmental factors. Various models for complex traits have been proposed [5], especially for epistatic interactions without major effects of individual loci [6].

It has been well recognized that DNA contains the information necessary for the development and functioning of organisms, and this information is contained in the sequence of its building blocks, nucleotides. For example, transcription factors (TFs) are proteins that can modulate the activity of these genes by binding to specific nucleotide sequences (binding sites) in or near genes [7]. Some of the early approaches to the detection of these sequence motifs were based on discrete discriminant analysis and scan statistics [8,9]. A state-of-the-art method is based on the machine learning algorithms [10]. DNA sequencing has facilitated disease diagnosis and prenatal diagnosis of chromosomal trisomies and has implications for precision medicine [11].

Recently, the field of FPM is rapidly emerging [12]. Most FPM methods can be represented as unsupervised approaches [12]. That is, the data are not tagged or labeled to belong to different classes, whereas the FPM approach can also be represented in a supervised setting [13]. The simplest case is that of two classes, for example, *cases* and *controls*. A straightforward implementation considers class labels just as additional items in the database; in a resulting association rule, *R: x*→ *y*, the right-hand side *y* would then represent the class label while *x* is a pattern of interest [13]. The *confidence* of *R* is given by the conditional probability, *P*(*y*|*x*), and its support is defined as P(x), that is, the expected proportion of the *x* pattern in the database. Based on the well-known *Apriori* algorithm [14,15], FPM seeks to find all patterns (sets) of items occurring with a minimum frequency (*support*) in a possibly large dataset. For example, supermarkets keep databases of items purchased by consumers. A set of items, X = “bread – butter – milk” represents such a pattern of groceries, and the number of individuals purchasing X is referred to as the support for X. Also, given a pattern, X, it is of interest to find an item (or another pattern), Y, with high conditional probability of occurrence. That is, the *confidence, k* = P(Y|X), indicates the likelihood that consumers purchasing X will also purchase Y. In many FPM implementations [1], searches are restricted to patterns with given minimum support and minimum confidence.

We previously exploited FPM methodology in genome-wide disease association testing [16,17], where each individual is assigned a genotype at each of a large number of DNA variants, and two classes of individuals exist; cases are those affected by a heritable disease and controls are unaffected. Classically, we would test each variant to see whether a genotype at that variant is associated with disease, that is, shows higher frequency in cases than controls. For (digenic) traits under the influence of two variants, possibly located on different chromosomes, we would like to find pairs of genotypes, with each member of a pair being from different variants, that show high frequency in cases. In FPM terminology, we are looking for patterns X of genotypes with high confidence, *k* = P(Y = “case” | X). Clearly, the higher the confidence, the more predictive a pattern is for disease. If a pattern occurs only in cases and not in controls, then the observed confidence is equal to 100%.

Many methods have been developed to make use of FPM approaches for detecting pairs of genotypes underlying digenic traits, or longer patterns of genotypes influencing polygenic traits. Here, we focus on practical ways of establishing whether genotype patterns found by such methods are statistically reliable.

## Selected Special Aspects of FPM

Most data contain errors and possibly missing data. Various approaches to handling the latter problem have been discussed [18], but in practice, it has been almost inevitable that some observations or variables must be deleted. For example, when patterns of genotypes are mined for their association with disease [17], preprocessing the data may involve deleting either individuals or DNA variants with a proportion of missing genotypes exceeding some threshold, whichever is more tolerable. More sophisticated approaches have been proposed in the form of noise tolerant item sets called *approximate frequent itemsets* (AFIs) [19], which obviate the need for deleting data but are computationally more demanding than frequent itemset mining (FIM) methods in error-free data. Newer methods for mining AFIs have been developed and show excellent computational properties [20].

Classical FIM methods are designed to find *frequent* patterns. However, patterns with properties other than being frequent may be more interesting. Various measures of interestingness have been discussed [21] and two will be mentioned here. (1) One specific property of patterns is their statistical significance—in general, it will be of interest to know whether a frequent pattern may be frequent by chance or whether there is more to it than randomness. Thus, mining significant patterns is of importance [22]. This is often achieved with some form of permutation analysis [23-25]. (2) In microarray data, mining frequent gene regulation patterns is an important task, but resulting patterns should show high utility; a corresponding utility model has been proposed that considers both the gene-disease association and the degree of expression level [26]. A survey of high utility itemset mining has recently been published [27].

Last but not least, sets of genotypes from DNA variants can be used for individual identification. For example, support vector machines (SVM) and random forest (RF) methods have been applied to mitochondrial DNA for identifying relatives of individuals who died in accidents [28]. Specialized approaches have been developed for finding sets of DNA markers, so-called Ancestry Informative Marker (AIM) systems, that can identify ethnic origin of individuals [29,30]. The basic characteristic for a DNA variant to serve as an AIM is that it has very different allele frequencies in different populations. For example, a specific allele at marker rs1876482 on chromosome 2 has frequency of 0.83 in a sample of 48 Japanese yet has not been observed in 34 Sardinians (Italy) and in 77 Yoruba individuals (West Africa) [29].

A well-known example of individual identification is that of the Kennewick Man, a human skeleton discovered in the American state of Washington, dated to have lived 9,000 years ago [31]. Controversies ensued between native Americans and scientists as to the ethnic origin of Kennewick Man. As a native American, he should be properly buried but as a non-native individual he should be available to further scientific study. Sequencing his genome revealed that he was indeed closely related to modern native Americans [31].

A recent example of relationship identification refers to Sitting Bull, the legendary Lakota Sioux leader [32]. Genomic analysis of a small piece of Sitting Bull’s hair confirmed that Ernie LaPointe of South Dakota USA is Sitting Bull’s great-grandson. It is also noteworthy that this genomic analysis revealed that Ernie is pure native American, unadmixed with Western genes.

## Multifactor Dimensionality Reduction

Among many FPM methods, multifactor dimensionality reduction (MDR) has been widely used for detecting epistasis [33]. For binary phenotypes of cases and controls, MDR finds the optimal interaction pattern that best predicts the disease status by dividing high-dimensional genotype combinations into a one-dimensional variable with high-risk and low-risk groups. The division is according to whether the ratio of cases to controls exceeds a threshold. The k-fold cross-validation was adopted to avoid overfitting. As evaluation measures, balanced accuracy and cross-validation consistency were used [34]. Since MDR is a kind of FPM model, it has several advantages compared to the conventional epistasis approach. MDR greatly reduces the dimensions of the data are effectively reduced. MDR does not assume any specific genetic model. MDR can easily identify high-order interactions even without significant main effects [35,36].

Since its introduction of MDR, many extensions of MDR have been proposed. For categorial traits, log-linear models MDR was proposed using log-linear models [37]. Robust MDR have been proposed to handle outlying observations [38]. Using the odds ratio, OR-MDR was proposed, replacing the naïve classifier with a more quantitative measure [36] and optimal MDR replaced the fixed threshold with a data-driven threshold using an ordered combinatorial partitioning algorithm [39]. For dealing with covariates of interest, generalized MDR (GMDR) was proposed based on the generalized linear models (GLMs) [40]. Since it was based on GLMs, GMDR can handle both dichotomous and continuous phenotypes. For dealing with continuous traits, quantitative MDR (QMDR) was proposed by comparing the sample mean of each genotype combination with the global mean [41]. To handle outliers and to make the distributional assumption free, cluster-based MDR has been proposed [42]. For survival time with censored data, Surv-MDR was proposed, which uses the log-rank test statistic as a classifier [43]. Later, Cox-MDR and accelerated failure time MDR were proposed for the survival phenotype based on Cox regression and the accelerated failure time model, respectively [44,45]. Recently, Kaplan-Meier MDR was also developed by using the Kaplan-Meier median survival time to define a classifier [46].

A multivariate version of MDR has been proposed to treat multiple phenotypes simultaneously. For example, obesity can be measured through body mass index, weight, and hip ratio. Multivariate generalized MDR (GEE-GMDR) can simultaneously address these obesity-related phenotypes by constructing generalized estimating equation models [47]. Extended multivariate QMDR was proposed using principal component analysis scores and Hotelling's T2 statistic as classifier and evaluation measure [48]. More recently, multivariate cluster-based MDR (multi-CMDR) has been proposed [49]. Multi-CMDR applies fuzzy k-means clustering to separate high-risk from low-risk groups and uses Hotelling's T2 statistic for evaluation. More recently, multivariate rank-based MDR (MR-MDR) was proposed as a new non-parametric multivariate approach based on a rank statistic for identifying genetic interactions. As in multi-CMDR, MR-MDR utilizes the fuzzy k-means clustering analysis with a noise cluster [50].

## Machine Learning Approaches

Classical statistical methods like discriminant analysis can classify data into two or more groups based on a possibly large number of input variables but they do this in a predictable manner, that is, with the aid of a clearly defined model. Machine learning methods improve their performance with experience [10]; for example, they “learn” what DNA sequences represent binding sites for TFs [10]. Differentiating statistical from machine learning methods is somewhat arbitrary [18,51]. Typical examples of machine learning algorithms are artificial neural networks (ANNs) [10], SVMs, and random forests [52]. Okazaki and Ott [53] provided a good review on machine learning approaches to digenic inherence. Here, some examples in genomics from each of these methods are cited.

One of the earliest applications of ANNs in human genetics was to find patterns of genetic loci that would discriminate between two phenotypes, affected versus unaffected with a complex genetic trait [54]. An ANN consists of “neurons” organized into layers connected with each other by various links. Signals received at the input layer will be transmitted to a hidden layer and eventually reach the output layer. Based on a dataset with known outcomes, the ANN “learns” through repeated training how best to predict outcomes. The simplest ANNs are equivalent to discriminant analysis models, but newer ANNs consist of multiple hidden layers of neurons resulting in “deep” learning [10]. Analysis of the DNA sequence provides many opportunities for the application of ANNs. For example, modern ANNs are much better at recognizing transcription factor binding sites than the earlier approaches mentioned above [10]. As another recent example, based on chest X-ray images, deep learning methods were successfully applied to classify individuals into one of three groups: coronavirus disease 2019 patients, healthy controls, and individuals with pneumonia [55].

An SVM is another machine learning algorithm that is generally applied for separating data into two groups based on input data related to these groups. This is accomplished by the construction of a hyperplane that best separates the two groups [56]. For example, SVMs were applied for finding DNA variants and their interactions that discriminate between cases and controls in Parkinson disease [57]. More recently, the SVM approach was considered the best method for diagnosing coronary diseases [58].

In the RF method, multiple classification trees are grown, which then “vote” on the best classification or prediction. An interesting comparison between the logistic regression and RF method was carried out in a retrospective study on 505 children receiving chemotherapy and had developed febrile neutropenia [59]. The two methods were applied to predict, which of these children would develop blood stream infection, which had occurred in 106 of the 505 children. Predictor variables included demographics and clinical and laboratory measures on initial presentation. As assessed by the receiver operating characteristic area under the curve, the RF model did better (0.79) than logistic regression (0.65). While the latter method models the dependency between predictor variables and outcome in a linear fashion, RFs allow for complex non-linear relationships [59].

Another, much larger study applied RF analysis to compare 56 risk/protective factors for depression in a sample of 67,603 European older adults [60]. Social isolation and poor health turned out to be the strongest risk factors, accounting for 22% of variability in depression.

## Statistical Significance and Discovery

Consider a number N of individuals, each genotyped at a possibly large number of DNA variants. For each variant, a given individual has two alleles numbered 1 and 2 (or 0 for unknown), which are conveniently translated into three genotypes numbered 1 = (1, 1), 2 = (1, 2), and 3 = (2, 2), where (*i, j*) refers to the set of two alleles. For two variants, possibly located on different chromosomes, there will be nine possible genotype patterns (pairs of genotypes). For example, the pattern (3, 2) refers to genotype 3 at variant 1 and genotype 2 at variant 2.

Any method for finding genotype patterns associated with disease will furnish a list of patterns, each with observed values for support *s* and confidence *k*. For a given pattern, X, the number of individuals may be displayed in a 2 × 2 table as shown in Table 1. A common statistic to measure association of X and Y is Pearson’s chi-square, *X*^2^, and its associated nominal empirical significance level, *p*, where *p* is the probability that chi-square is as large as *X*^2^ or larger just by chance, that is, assuming no association between X and Y. We generally want to find results with an associated very small *p*-value such that we are confident that the result could not have been obtained by chance alone and is in fact due to an effect of X on disease.

Here we want to shed light on questions on multiple testing in genotype pattern mining (GPM) for case-control association analysis of digenic traits. For statistical details, the reader is referred to published reviews [61,62].

## Bonferroni Correction

Assume now that we have obtained a number, *m*, of chi-square results and associated nominal (raw) p-values, *p*_i_, *i* = 1…, *m*, each obtained for a suitable genotype pattern (support and confidence larger than specified minimum values), where *m* can range from a few dozen to many millions. Because they are based on pairs of variants with a given variant possibly participating in multiple pairs, these p-values are likely correlated.

The Bonferroni method controls the so-called family-wise error rate (FWER) by declaring *p*-values significant only if *p*_i_ < α/*m*, where α is the overall rate of false positive results. This result can be rephrased in terms of Bonferroni-adjusted p-values, *p*_i_^B^ = min(*m p*_i_, 1) that are valid for any dependence among p-values, although the Bonferroni correction tends to be conservative for strongly correlated p-values [61].

Researchers often search for patterns with high confidence, and such patterns are highly likely to furnish large chi-squares. In other words, results are biased in favor of large chi-square values. The best remedy is to relax the selection procedure as follows. Observed confidence without any disease association is given by the proportion of cases in the data, that is, by *k*_0_ = *N*_2_/(*N*_1_ + *N*_2_), where *N*_1_ and *N*_2_ are the numbers of controls and cases, respectively. Thus, our search criteria should impose a minimum confidence of *k*_0_ rather than the usual 80% or 90%, which will guarantee that the full range of p-values from 0 through 1 will be exhibited. In practice, this will often lead to very large numbers of patterns, which is the price we pay for applying a Bonferroni-type multiple-testing correction. As will be seen in our practical examples, we may not need to rigorously apply a minimum confidence of *k*_0_ and working with the Bonferroni correction turns out to be rather reasonable.

Various approaches have been taken to improve on the basic Bonferroni approach. For highly interrelated tests, an early suggestion by Tukey [63] is to use *m*´ = √*m* instead of *m* in the Bonferroni correction. Depending on the specific data analyzed, it may be possible to identify some patterns as being unobservable, which can lead to a number *m*´ of tests considerably smaller than *m*, that is, the multiple testing burden is alleviated to some degree [64]. A method directly estimating the correlation between tests and thus deriving a number *m*´ of effectively independent test was developed some 20 years ago [65] but, despite its elegance, it does not seem to have been applied very often.

At this point it is worth mentioning that the practice of working with a minimum confidence will lead to one-sided statistical tests, that is, we only consider patterns more common in cases than controls. We may reverse the pattern search by looking for patterns more common in controls than cases, or apply other criteria for selecting patterns. But here we focus on the common practice of restricting patterns to those with high confidence.

## Permutation Testing

As we have seen, for the Bonferroni method to provide valid results, we should consider the whole range of p-values from small to large, effectively comparing frequencies of small and large p-values. For example, without any effect of genotype patterns on disease (called null hypothesis), the proportion of patterns with p < 0.10 should be the same as those with p > 0.90, namely, 10%. Another type of comparison may be performed by creating null data on the computer, that is, data known not to contain disease association, and comparing these with the observed data. Null data may be obtained by randomly scrambling labels “case” and “control”, which clearly gets rid of any association between genotypes and phenotypes, whereas the genotype data are left untouched. A large number Np of permutation datasets are created on the computer, with each being subjected to the same analysis as done on the observed data, and the *largest* test statistic, *T*_i_ (here, chi-square), obtained for each such null dataset is recorded. Then, for each chi-square obtained in the observed data, the proportion of *T*_i_ values larger than or equal to the observed chi-square is an estimate for the *p*-value associated with that observed chi-square [66]. Permutation testing has been carried out in many areas of research [67]; specific improvements in the data mining area have recently been described [24].

The main advantage of permutation testing is that a potentially small number of highly selected genotype patterns (high values for minimum confidence) may be considered and their permutation-based *p*-values obtained. On the other hand, for each null permutation, the whole process of searching for genotype patterns must be repeated and this should be done at least 1,000 times.

## False Discovery Rate

The methods discussed so far control the so-called FWER [61] by determining the probability p that a result as extreme or extremer than the one observed could have been obtained by chance alone. If a p-value is smaller than a limit like 0.01, then a result is called *statistically significant*. A different approach to this situation is to focus on significant results and ask, what proportion of them is false? This proportion is called the false discovery rate [68], false discovery rate (FDR), and results with FDR smaller than some limit like 0.01 are called *discovered*. Several methods for determining the FDR have been described; the most reliable, and most conservative, of these is the Benjamini-Yekutieli method [69]. As with the Bonferroni correction, we need to consider the whole range of p-values, that is, a potentially very large number *m* of genotype patterns with minimum confidence of *k*_0_ = *N*_2_/(*N*_1_ + *N*_2_). The p-values are ranked from small (*p*_1_) to large (*p*_m_), and the largest *p*_r_ < *r* × α/[*m* × c(*m*)] is determined, where *r* is the rank and c(*m*) = 1 + 1/2 + 1/3 + … + 1/*m* is the harmonic series. All values of *p* < *p*_r_ are considered discovered [61].

The idea underlying the FDR has been formulated as a partition test [70] and may lead to more discoveries than FDR [71], but this approach has not been pursued further. In our experience, the number of discovered patterns is often similar to the number of patterns deemed significant after Bonferroni correction. In fact, if the smallest *p*-value is significant then it is also discovered [68].

## Results

For some datasets, the total number of variants may be too large for pattern mining approaches. Some authors then select a subset of variants, often those *N* variants with the largest single-locus disease association [72]. However, this practice is fallacious as a strong disease association of a variant has the effect that it will show up in many genotype patterns. In other words, a genotype pattern may show a strong disease association mostly because of main effects of one or the other of the two participating variants rather than an interaction effect [17]. If the aim is to uncover interaction effects, the recommended approach is to first *remove* individually significant variants and only then proceed to genotype pattern analysis.

As a case in point, we apply our GPM approach [17] to a published dataset on wet age-related macular degeneration collected in Hong Kong [73] and analyze results for significance and discovery. This dataset contained 96 cases and 127 controls, each genotyped for 81,934 variants genome-wide. Single-variant analysis with the trend test as implemented in plink indicated two variants with permutation-based p < 0.05. A total of four variants had permutation-based p < 0.60 and were removed for digenic analysis so that virtually no variants with appreciable main effects were left.

GPM analysis was carried out with minimum support of 40 digenic genotype patterns. The “null” confidence was 96/223 = 43%, but to keep the number of patterns to a manageable level, we were working with a minimum confidence of 60%, which resulted in a total of 18,044,794 genotype patterns. Permutation analysis was carried out with 1,000 replicates. Table 2 shows the number of significant or discovered patterns as determined by methods discussed above.

Results for the Bonferroni correction are comparable to those for FDR-BY and furnished more patterns than permutation analysis. Thus, at least on the basis of this example, the Bonferroni correction is a reasonable way to go although, to be valid, it requires a rather low minimum confidence with resulting large number of patterns. Despite the rather large total number of genotype patterns and resulting strong penalty, the Bonferroni correction exhibited multiple significant genotype patterns. Permutation analysis provided much smaller number of significant results. However, it is not generally true for most cases. Depending on the number of replicates, the permutation analysis may provide slightly different results.

## Discussion

Pairwise analyses may be carried out at the level of genotype, variant [74], or gene [75], with respective decreases in granularity and total numbers of pairs. Genotype pairs (patterns) offer the greatest precision as signals of a single genotype pair may be hidden among the nine genotype pairs in a pair of variants [17]. On the other hand, searching among very large numbers of genotype pairs requires good computer resources. It is hoped that developments in search methodology and computer programming will improve FPM methods in genotype patterns mining and allow for permutation analysis with suitable numbers of replicates.

As seen in the results displayed in Table 2, a small number of genotype patterns are significant even in the absence of significant single variants with strong main effects (the significant ones were removed prior to analysis). This phenomenon can be expected to be encountered more often in the future when FPM methods become more powerful and more generally available even on smaller computers.

## Figures and Tables

**Table 1.** Layout of individuals for a given genotype pattern, X

	No. of individuals		
Phenotype, Y	With X	Without X	Sum
Affected, “case”	*a*	*b*	*N*_2_
Unaffected, “control”	*c*	*d*	*N*_1_
			

Observed support is given by *s* = *a* + *c*, and confidence is *k* = *a*/(*a* + *c*).

**Table 2.** Number of significant (permutation, Bonferroni) or detected (FDR) genotype patterns for given overall significance level, α

α	Permutation analysis	Bonferroni correction	FDR-BY
0.001	0	2	2
0.01	0	9	3
0.02	2	11	11
0.03	2	16	13
0.04	2	18	61
0.05	2	19	11905

FDR-BY refers to the false discovery rate by the Benjamini-Yekutieli method; the result for α = 0.05 seems unreasonable.
